# The Impact of Hydration and Dehydration on the Mobility and Location of Ibuprofen Molecules in the Voids of Ultra-Stable Zeolite Y

**DOI:** 10.3390/ma14247823

**Published:** 2021-12-17

**Authors:** Mariusz Gackowski, Mateusz Paczwa

**Affiliations:** 1 Jerzy Haber Institute of Catalysis and Surface Chemistry, Polish Academy of Sciences, Niezapominajek 8, 30-239 Krakow, Poland; 2 Institute of Physics, University of Szczecin, 70-451 Szczecin, Poland; mateusz.paczwa@usz.edu.pl

**Keywords:** mesoporous zeolites, confinement effect, guest-host systems

## Abstract

Mesoporous dealuminated zeolites are used as hosts for ibuprofen. This drug experiences high mobility when confined in mesopores, which is largely dependent on the water content. Zeolites are materials that are naturally hydrated under ambient conditions. Nitrogen adsorption and X-ray diffraction (XRD) show that the samples with the content of ibuprofen up to 38% have the guest phase residing only in mesopores. ^1^H and ^13^C MAS NMR studies of samples in ambient conditions, after dehydration, and in hydration prove the impact of water for increased mobility of ibuprofen. Increased mobility of the introduced phase was also detected for samples with no water content. It was ascribed to ibuprofen located outside mesopores, which experiences a prolonged time of cooling to room temperature. This phenomenon is important for all the future uses of the melting method in guest–host systems and the future use of zeolites for biomedical applications.

## 1. Introduction

Molecules in confinement experience different physico-chemical properties from those in bulk [1,2,3]. In some cases, molecules located in confined space do not form crystals and thus can demonstrate improved dissolution and bioavailability [4]. Moreover, molecules in confinement can experience shifted temperatures of phase transitions, e.g., freezing and melting temperatures [5]. Most of the studies focused on an introduction to pores of different host material chemicals that are liquid in ambient conditions, and their freezing, melting, and glass transitions were examined [5,6,7,8,9,10,11,12,13,14]. Many organic and inorganic guest molecules have been used, with water being probably the most widely studied [7,9,10,12,13,14].

Ibuprofen is a propionic acid derivative belonging to the class of nonsteroidal anti-inflammatory drugs. It has been widely studied in different drug delivery systems, being introduced in vessels based on silica [15,16,17,18,19,20,21,22,23], polymers [24,25,26], and carbons [27,28], among others. Bulk ibuprofen is a crystalline solid in ambient conditions, but it exists in a different state when it is introduced into mesopores. It experiences a specific confinement effect as well. It exhibits high mobility, a glassy-like or even liquid-like state when it is introduced into a mesoporous MCM-41 (Mobil Composition of Mater no. 41) material [29]. It was shown that ibuprofen exhibits higher mobility in larger pores (116 Å), in comparison to smaller ones (35 Å). Due to this specific dynamic behaviour of ibuprofen in confinement, it was possible to use NMR (nuclear magnetic resonance) techniques characteristic for liquid-state NMR (like INEPT (insensitive nuclei enhanced by polarization transfer)) to characterize solid samples.

The importance of water in the ibuprofen–mesoporous silica system has been suggested in the literature. By the means of DFT (density functional theory) calculations it was showed that water plays an important role in the mobility of ibuprofen introduced into microsolvated MCM-41 material. It was proven that water interacts with ibuprofen by “dynamical” H-bonds: the organic molecules desorb and readsorb on the microsolvated surface of the silica, therefore obtaining high mobility [30]. In ref. [31], it was stated that apart from the confinement effect, a chemical interaction between the organic molecule and the surface of the host also impacts the dynamical behavior of the guest molecules. By means of variable temperature NMR, it was proven that a fast chemical exchange between the COOH group of the drug molecules and water molecules is taking place. Using a ^1^H-^29^Si-^1^H “double back” CP (Cross Polarization) experiment, the signal from water H-bonded to silanols was identified. Potrzebowski et al. [32] showed that water vapour can push the ibuprofen out of mesopores. Recently, SBA-15 (Santa Barbara Amorphous-15) samples with ibuprofen introduced into mesopores using the melting method were examined [33]. SBA-15 is a highly stable mesoporous silica-based material with ordered hexagonal cylindrical pores and narrow pore size distribution. The samples have been examined in the ambient (hydrated) state and in a dehydrated state after using an oven or a vacuum treatment (both at 80 °C for 20 h). For a more complete dehydration, samples prepared under a N_2_ atmosphere after complete dehydration of the host in vacuum (at 300 °C in 20 h) have been prepared. It was evident that water was crucial for the existence of high mobility ibuprofen, while it exhibited decreased mobility in a dehydrated state. The presence of water also changed the location of ibuprofen molecules (inside or outside mesopores). The kinetics of the release of ibuprofen to simulated body fluid (SBF) showed a correlation with a higher mobility of introduced phase inside mesopores, exhibiting the faster release of hydrated samples, in comparison to non-hydrated ones.

Zeolites are aluminosilicates that come with more than 200 different topologies, i.e., different sizes, shapes of pores (in the range on micropores), and spatial connections between them. This creates a wide range of spatially different materials with very high surface areas. In the last years, a growing interest in the field of hierarchical zeolites can be noticed. These are zeolites that apart from the standard micropores also possess a second type of pores in the range between 2 and 50 nm (mesopores). These two generations of pores are interconnected. It is one of the strategies to fight with limited diffusion in zeolites and make the acid sites located in zeolites accessible to bulky molecules. There are many strategies to obtain mesoporous zeolites. One of the most widely used zeolites in the industry, ultra-stable zeolite Y used in fluid catalytic cracking (FCC), has mesoporosity generated by steaming and acid treatment. It is easily accessible or synthesized and thoroughly investigated in the literature, and therefore makes a perfect choice for the study of the use of zeolites as hosts for medically active compounds.

Zeolites are preferably used as sorbents [34] and catalysts [35,36,37], but also in biomedical applications [38,39,40,41]. They have been used as drug delivery systems of many drugs (ibuprofen, fluorouracil, sulfadiazine, and others). Ibuprofen molecule dimensions (ca. 11 Å × 8 Å × 5 Å) make it more preferential to be introduced into mesoporous materials with pore widths from 5 to 50 nm, rather than solely microporous materials, like standard zeolites. Horcajada et al. used mesoporous zeolites Y with different Al content to introduce ibuprofen molecules [42]. These materials exhibited mesoporosity created by a dealumination process. They discovered that the amount of ibuprofen adsorbed into the material is related to the volume of pores and is comparable to this obtained for pure-silica MCM-41 material. The ibuprofen release rate was connected with Al atoms in the structure: the presence of extra-framework Aluminum and the Si/Al ratio of framework atoms.

Zeolites are known to be perfect sorbents for water, which is directly connected with framework aluminum [43]. It is also known that mesoporous zeolites exhibit enhanced water diffusivity in comparison to standard zeolites [44,45]. Therefore, mesoporous zeolites are perfect candidates to deepen insights concerning the impact of water on the dynamic behaviour of ibuprofen molecules in confinement. Here, the study on the incorporation of ibuprofen molecules into mesoporous ultra-stable zeolite Y is presented. The aim of this study was to verify if the mobile, liquid-like behavior of ibuprofen molecules observed for mesoporous silicate materials is also observed for mesoporous zeolites, as well as to check how water naturally occurring in zeolites changes the characteristics of ibuprofen in confinement. To do that, samples under atmospheric humidity, additional hydration, and in dehydrated states were studied. Nitrogen adsorption was used to study the changes in porosity after the introduction of ibuprofen. X-ray diffraction (XRD) was applied to check if ibuprofen crystallizes in this system, while thermogravimetry (TG) and differential scanning calorimetry (DSC) showed the content of ibuprofen in the samples, and the phase changes of the organic molecules under confinement. Magic angle spinning nuclear magnetic resonance (MAS NMR) was applied to characterize the samples under different states of hydration. This is probably the first study to utilize the solid-state NMR technique to study the effect of water on the dynamic behaviour of ibuprofen introduced into mesoporous aluminosilicates. The results could have a significant impact on the future use of this system in drug delivery applications.

## 2. Materials and Methods

Dealuminated zeolite denoted as FAU31 was purchased from Zeolyst (Kansas City, MO, USA) (CBV760). Ibuprofen (98%) was purchased from Sigma-Aldrich (Darmstadt, Germany).

Samples denoted as FAU31-12IBU, FAU31-24IBU, and FAU31-38IBU were synthesized by mixing 300 mg of zeolite powder with 30 mg, 90 mg, and 150 mg of ibuprofen, respectively. Afterwards, samples were kept in an oven heated to 85 °C for 1 h. After at least 20 min or more of equilibration at room temperature, they were transported to further examination.

For studies of hydration and dehydration, the samples were kept in a desiccator with 49% of humidity over a saturated solution of Mg(NO_3_)_2_ for 19 h or in an oven heated to 85 °C for the same time, respectively.

The samples synthesized in a water-free environment were prepared using a degassed zeolite: 0.15 mg of a zeolite sample was dehydrated in a vacuum line at 300 °C or 500 °C for 19 h, after which a glass tube with the sample was sealed. It was transported to a glove box with N_2_ gas. Inside, a glass tube with the degassed sample was opened and mixed with ibuprofen. After that, a rotor with the sample was transported to a vacuum line using a glass device with a Teflon valve. In the Schlenk line, a thermal treatment was applied (85 °C for 1 h). After this, a glass tube was opened in a glove box, where a rotor was closed and transported to NMR spectrometer (Bruker BioSpin GMBH, Rheinstetten, Germany).

X-ray diffractometry (XRD) results were obtained using a PANalyticalCubixX’Pert Pro diffractometer (PANanalytical, Almelo, The Netherlands), with CuKa radiation (lambda = 1.5418 Å) in the 2theta angle range 5–50°. The N_2_ sorption at 77 K was performed on ASAP 2420 Micromeritics apparatus (Micromeritics, Norcross, GA, USA). Prior to the adsorption, samples were degassed in a vacuum at 298 K for 18 h. The specific surface area has been determined using the nitrogen adsorption method. Pore size distribution has been calculated using a BJH method from a desorption branch. Thermogravimetric analysis (TG) and differential scanning calorimetry (DSC) curves have been recorded using Netzsch STA 409 PC Luxx (Netzsch, Selb, Germany) in the temperature range from 30 to 1000 °C (10 °C min^−1^).

^1^H and ^13^C MAS (magic angle spinning) NMR spectra were recorded using Bruker Avance III 500 MHz spectrometer (Bruker BioSpin GMBH, Rheinstetten, Germany) with frequencies 500.1 and 125.8 MHz, respectively, and RF fields of 60 kHz. Typically, for ^1^H MAS NMR spectra a single pulse π/2 excitation and recycle delay of 10 s were used. Typically, 32 scans were collected. For ^13^C CP MAS spectra, 2 ms of contact time, 70–100% of camp amplitude, and SPINAL64 decoupling were used. The same decoupling sequence was used for ^13^C HPDEC (high power decoupling) MAS NMR experiments. Typically, 256 scans were collected for ^13^C CP and 410 scans for ^13^C HPDEC MAS NMR spectra. All chemical shifts were referenced to TMS (tetramethylsilane). If not stated otherwise, 4 mm rotors were used to spin the samples to 10 kHz.

^1^H MAS NMR spectra in a temperature range from −50 to 45 °C were performed on Bruker Avance III 400 WB spectrometer (Bruker BioSpin GMBH, Rheinstetten, Germany) (*B_0_* = 9.4 T) with a 4 mm probe and a radio frequency field 60 kHz in the Laboratory of Radiospectroscopy of University of Szczecin. Samples were spun at the magic angle using ZrO_2_ rotors and ZrO_2_ caps at MAS frequency of 10 kHz. For temperatures below 270 K, we used a heat exchanger to produce the cooling gas. ^1^H MAS NMR spectra were recorded by a single pulse π/2 excitation and the recycle delay of 5 s by 10 scans.

## 3. Results

### 3.1. Synthesis and Characterization under Ambient Conditions

Table 1 shows the content and porosimetric characteristics of synthesized samples. While the content of ibuprofen increases, the content of water decreases. The specific surface area (S_BET_) and pore volume decreases as well, from 716 to 33 m^2^/g and 0.53 to 0.01 cm^3^/g, respectively. Diameters of pores do not change. Pore size distributions depicted in Figure S1 (Supplementary Information) show these trends graphically. In the sample FAU31-38IBU, there is no detectable volume of pores indicating that all pores are filled with guest molecules. Another possibility is that ibuprofen located outside mesopore clogged the entrance to the pores hindering the penetration of the pores by N_2_ molecules.

**Figure 1:**
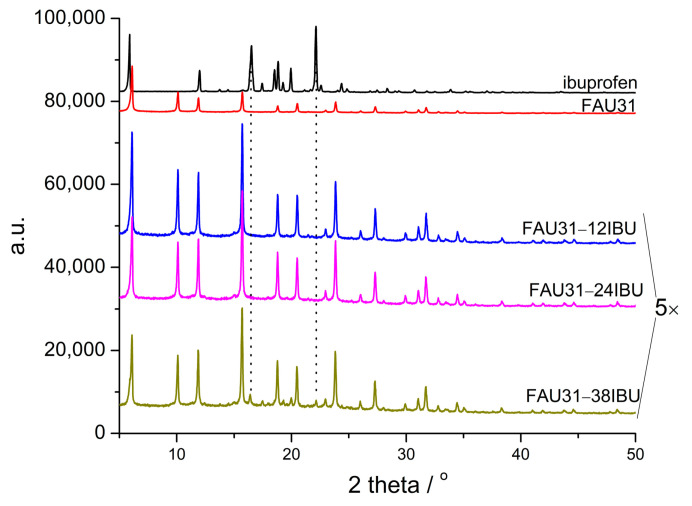
XRD diffraction patterns of the samples under study. For a clearer picture, patterns signed ‘5×’ are magnified 5 times.

Figure 1 shows XRD patterns of the samples under study. The diffraction pattern characteristic for faujasite is preserved after the introduction of organic molecules [46]. There are no detectible diffraction peaks for ibuprofen in most of the samples. Only the sample with the highest content of ibuprofen exhibits very low intensity diffraction peaks characteristic for the guest phase. It means that only in this sample, a small part of ibuprofen forms crystals but the majority of it, as in other samples, is present in a non-crystalline form.

**Figure 2:**
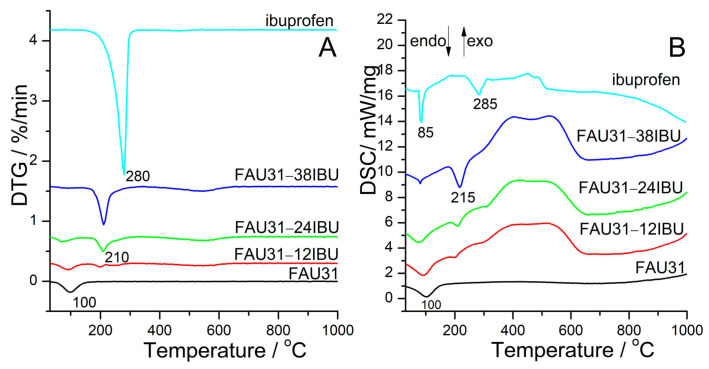
DTG (**A**) and DSC (**B**) curves of the samples under study.

Thermogravimetric analysis (TG) and differential scanning calorimetry (DSC) provide an insight into temperatures of phase changes that ibuprofen experience upon confinement. TG curves are showed in Supplementary Information (Figure S2). Differential thermogravimetric (DTG) and DSC curves depicted in Figure 2 show that there is a shift of the evaporation temperature of ibuprofen located in mesopores of FAU31 with respect to bulk molecules. It shifts from ca. 280 °C to 210 °C (∆T = 70 °C), and therefore the shift is higher than for SBA-15 (∆T = 55 °C) [33]. The melting temperature of ibuprofen can be detected as an endothermic peak in the DSC curve. For bulk ibuprofen, it is 85 °C, which is in agreement with the literature [47]. In most of the samples, this signal overlaps with the signal from water that is present at 100 °C and is exclusively seen in the host FAU31. Only in the sample FAU31-38IBU is there a small peak at 85 °C that could be discerned from a wider signal from water. Broad exothermic peaks in the region from 400 to 600 °C probably come from the oxidation of organic molecules.

**Figure 3:**
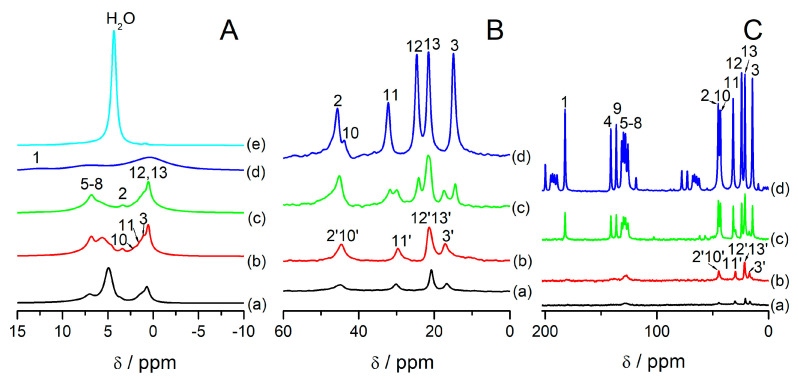
^1^H (**A**),^13^C HPDEC (**B**), and ^13^C CP (**C**) MAS NMR spectra of the samples: FAU31–12IBU (**a**), FAU31–24IBU (**b**), FAU31–38IBU (**c**), ibuprofen (**d**), and FAU31 (**e**) under ambient conditions (room temperature and atmospheric humidity). The signals were assigned according to the convention presented in Figure S3 (Supplementary Information). Apostrophes indicate signals from ibuprofen molecules located in mesopores (more details in text).

Figure 3 shows MAS NMR spectra of the samples under study. In Figure 3A, ^1^H MAS NMR spectra are presented. Signals are assigned according to the convention presented in Figure S3 (Supplementary Information). All the samples exhibit a higher resolution in comparison to bulk ibuprofen (Figure 3A(d)), but the resolution is not as high as in the samples with ibuprofen introduced to SBA-15 [33] or MCM-41 [29]. In the latter, the linewidths in ^1^H MAS NMR spectra was ca. 30 Hz, for SBA-15 ca. 100 Hz. Here, we cannot differentiate all the signals from protons. Specifically, the signals from protons 2, 10, 11, and 3 are not present as discernible separate peaks but overlap each other. It could be due to the fact that a part of the introduced ibuprofen is probably strongly bonded with the zeolite surface, which makes that part of the introduced phase less mobile. The signal from water is also present and its intensity is higher with the lower content of ibuprofen in the samples. ^13^C HPDEC (Figure 3B) and ^13^C CP (Figure 3C) MAS NMR spectra of the samples under study also show distinct changes in reference to bulk ibuprofen. They present signals characteristic for ibuprofen located in mesopores, which are highlighted with apostrophes in Figure 3B,C. These signals are used in this article as “fingerprints” of ibuprofen located in mesopores that exhibits high mobility. Namely:

- The signal 3′ which is the signal from carbon 3 shifted to lower field,
- The signal 11′ which is the signal from carbon 11 shifted to higher field,
- Signal 12′13′ which is due to overlapping the signals from carbons 12 and 13
- Signal 2′10′ which is due to overlapping the signals from carbons 2 and 10.

The overlaying of the signals is due to motional averaging due to the high mobility of confined species. These signals are the same as what was observed in the literature for ibuprofen confined in silica mesoporous materials [29,33]. ^13^C NMR spectra of ibuprofen in solution show the same signals [48]. Based on this assignment, it can be said that samples FAU31-12IBU and FAU31-24IBU exhibit signals from mobile ibuprofen located only in mesopores, while the sample FAU31-38IBU signals from ibuprofen located both inside and outside mesopores.

### 3.2. Hydration and Dehydration under Atmospheric Pressure

Owing to a known impact of water on the mobility of ibuprofen molecules in confinement, the experiments of hydration and dehydration on SBA-15 with introduced ibuprofen have been performed.

**Figure 4:**
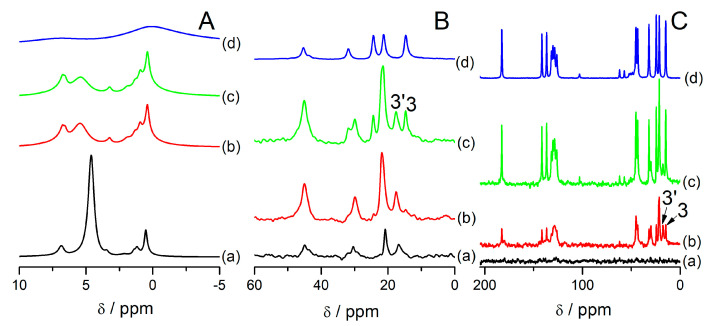
^1^H MAS NMR (**A**), ^13^C HPDEC MAS NMR (**B**), and ^13^C CP MAS NMR (**C**) spectra of the samples: FAU31–12IBU (**a**), FAU31–24IBU (**b**), FAU31–38IBU (**c**), and crystalline ibuprofen (**d**) after 19 h hydration in desiccator filled with Mg(NO_3_)_2_. All the spectra were normalized to the mass of the sample. For clarity, spectra in (**B**) (**d**) and (**C**) (**d**) were divided by the factor of 10. The signals were assigned according to the convention presented in Figure S3 (Supplementary Information). Apostrophes indicate signals from ibuprofen molecules located in mesopores (more details in text).

Figure 4 shows ^1^H (A), ^13^C HPDEC (B), and ^13^C CP (C) MAS NMR spectra of the samples after hydration for 19 h in a desiccator over a saturated solution of Mg(NO_3_)_2_. Crystalline ibuprofen was also hydrated this way, but its NMR spectra are not changed in respect to the same sample under ambient conditions (Figure 4A(d),B(d),C(d)). ^1^H MAS NMR spectra show higher signals from water and more distinctive peaks from protons 3, 10, and 11 in comparison to samples measured in ambient conditions (Figure 4A). This agrees with a previous study in that water increases the mobility of ibuprofen located in mesopores of SBA-15. It was shown that dehydrated or prepared in a water-free environment samples exhibited more broadened signals from ibuprofen in the ^1^H MAS NMR spectra with respect to the samples measured under atmospheric humidity, indicating the much lower mobility of the guest molecules [33]. ^13^C MAS NMR spectra show no changes in comparison to samples in ambient conditions. Only the ^13^C CP MAS NMR spectrum of FAU31-24IBU exhibits a signal from carbon 3 that was absent in the ambient condition (Figure 4C), which indicates that there are ibuprofen molecules located outside mesopores in this sample. According to other studies, the sample with the lowest amount of ibuprofen should have the highest mobility of drug molecules [29,33]. Indeed, the sample FAU31-12IBU has no detected (Figure 4C(a)) ^13^C CP signals, which could be due to the high mobility of those species. On the other hand, only this sample shows a lack of the signal from protons 3 in ^1^H MAS NMR spectra. This could be due to the interaction of ibuprofen molecules with the zeolite framework. Ibuprofen interacts with a zeolite host via hydrogen bonds of carboxylic groups with zeolite’s hydroxyl groups and water molecules, provided a hydrated state is present. There could also be another interaction, namely strong coordinative bonds between carboxylate species and extra-framework aluminum [42]. It was shown that in ultra-stable zeolite Y with a high content of extra-framework Al, the carbonyl group is deprotonated and FT-IR spectra show bands characteristic for monodentate carboxylic species. This interaction could immobilize at least the part of the molecule that is close to the carboxylic group, like a methyl group that protons 3 are a part of.

**Figure 5:**
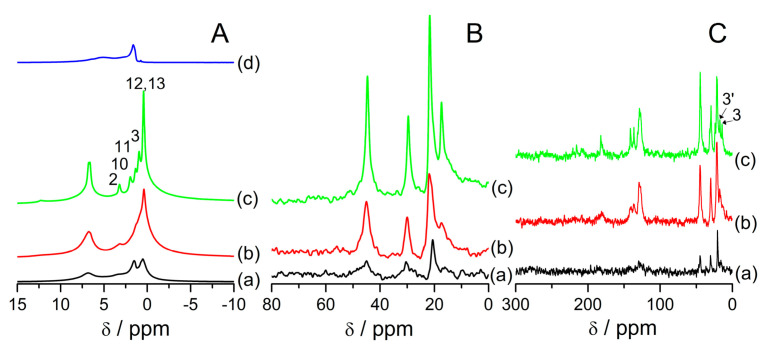
^1^H (**A**), ^13^C HPDEC (**B**), and ^13^C CP (**C**) MAS NMR spectra of the samples: FAU31–12IBU (**a**), FAU31–24IBU (**b**), FAU31–38IBU (**c**), and FAU31 (**d**) after dehydration in an oven for 19 h in 85 °C. The signals were assigned according to the convention presented in Figure S3 (Supplementary Information). Apostrophes indicate signals from ibuprofen molecules located in mesopores (more details in text).

Figure 5 shows MAS NMR spectra of the samples after dehydration in an oven at 85 °C for 19 h. Samples FAU31-12IBU and FAU31-24IBU exhibit almost no changes in reference to samples in ambient conditions in all the spectra, except the lack of water signals in ^1^H MAS NMR spectra. NMR spectra of FAU31-38IBU change distinctly. ^13^C HPDEC MAS NMR spectrum in Figure 5B shows no signal from carbon 3, so ibuprofen was located outside mesopores with low mobility. This signal is present in ^13^C CP MAS NMR (Figure 5C(c)), but the relative intensities of signals from carbons 3 and 3′ is similar, contrarily to samples examined in ambient condition, when the signal from ibuprofen located outside mesopores (carbon 3) was much more intense than the signal from carbon 3′.

The most resounding for the dehydrated sample FAU31-38IBU was the ^1^H MAS NMR spectrum, which showed a clear increase of the resolution with distinct peaks for protons 10, 11, and 3.

### 3.3. Synthesis in a Water-Free Environment

**Figure 6:**
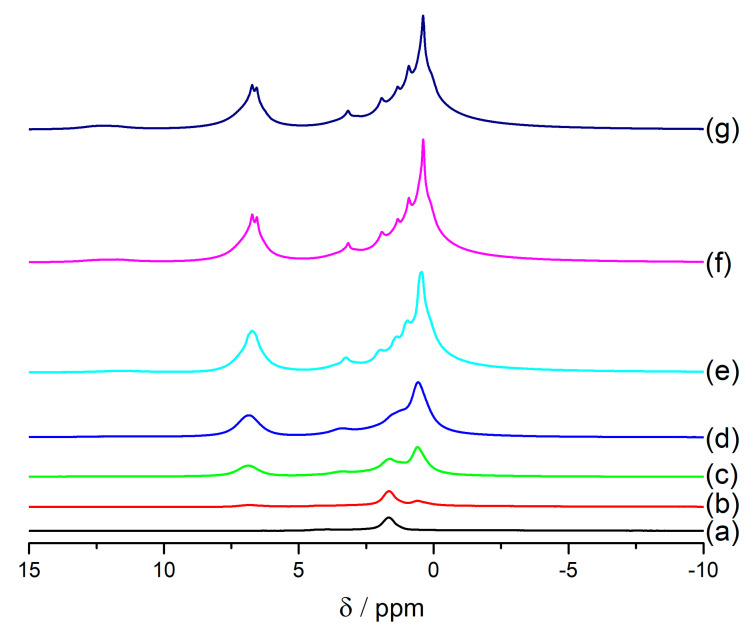
^1^H MAS NMR spectra of FAU31 sample after dehydration in vacuum at 300 °C (**a**) and samples containing: 3% (**b**), 9% (**c**), 15% (**d**), 34% (**e**), 46% (**f**), 66% (**g**) of mass % of ibuprofen in dehydrated zeolite. All the spectra were recorded in 295 K. Samples were measured using 3.2 mm rotors and with 12 kHz rotation.

To further examine this issue, a series of experiments in a dehydrated state have been done. For the samples synthesized under ambient conditions, water was present in the parent material, as well as in the samples with ibuprofen (Table 1). Samples dehydrated in an oven probably also contained some water, because the parent zeolite FAU31 exhibits signals from water in ^1^H MAS NMR (Figure 5A(d)). Here, the host material was dehydrated in a vacuum, ibuprofen was introduced in a water-free environment, and the heating treatment was performed in a vacuum, so that no water was present in the samples examined in NMR spectroscopy. Figure 6 shows ^1^H MAS NMR spectra of the samples prepared in a water-free environment with varying ibuprofen content, from 3% to 66% of ibuprofen in the samples. When the content of ibuprofen was 34% or more, there is a visible increase in the resolution of ibuprofen signals.

**Figure 7:**
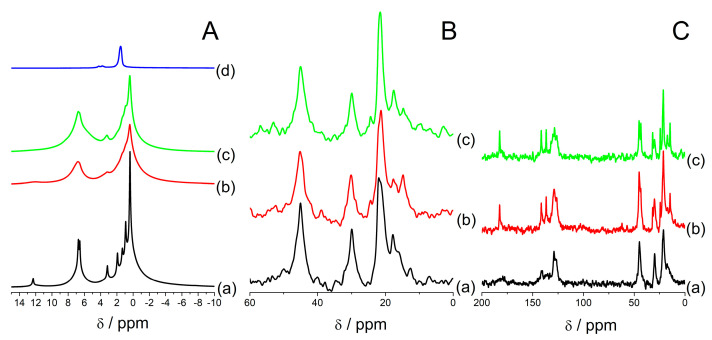
^1^H (**A**), ^13^C HPDEC (**B**), ^13^C CP (**C**) MAS NMR spectra of sample containing 28% of mass % of ibuprofen synthesized in water-free environment using vacuum heating at 85 °C: spectra after the synthesis (**a**), after equilibrating for 48 h in a closed rotor in a water-free disiccator (**b**), and after 4 h after opening the rotor to humid atmosphere (**c**). ^1^H MAS NMR spectrum in (**A**) (**d**) shows the parent zeolite FAU31 without ibuprofen dehydrated in vacuum at 500 °C. All ^1^H spectra are normalized to sample mass.

When a 4 mm NMR probe was used, an even higher resolution can be found, like this presented in Figure 7A(a). The high resolution of ^1^H MAS NMR spectra in a dehydrated state was maintained even after 3 h after the thermal treatment in vacuum (Figure S4, Supplementary Information). Moreover, the signals got even narrower this time. This sample exhibited ^13^C signals exclusively from ibuprofen of high mobility (Figure 7B(a),C(a)).

After that, a closed rotor was kept in a dehydrated state in a desiccator filled with silica for 2 days, and after this time, the spectrum of the sample changed. There is no high resolution of ^1^H MAS NMR spectrum. ^13^C MAS NMR spectra showed the presence of ibuprofen located outside mesopores (carbon 3) (Figure 7B(b),C(b)). Then, a rotor was opened for 4 h and measured in a partially hydrated state, which made small changes in the ^1^H MAS NMR spectrum (Figure 7A(c)), making signals a little narrower and presenting a small shoulder at ca. 6 ppm due to water molecules. ^13^C MAS NMR spectra in Figure 7B,C also only slightly changed.

### 3.4. High Mobility and the Effect of the Temperature

What is the reason for the observed temporary higher mobility in a dehydrated state? At first, it was assessed whether it is connected with a possible presence of residual water in the sample. The host material for the introduction of ibuprofen was dehydrated in vacuum at 300 °C, while 450 °C or more is used in many articles for complete dehydration of zeolites [49], and after activation at 350 °C, strongly bonded water is detected in hierarchical zeolite Y [50]. Indeed, there are some differences in ^1^H MAS NMR spectra of the samples dehydrated at 300 °C and 500 °C. Specifically, there are signals at ca. 6 ppm and a broad resonance at ca. 2.5 ppm that is present in the sample dehydrated using a lower temperature (Figure S5, Supplementary Information). These signals can be connected with the presence of strongly bonded water, the kind of water that is not desorbed at 300 °C. The signal at 2.5 ppm is connected with water interacting with SiOH groups [51], while the peak at ca. 6 ppm is connected to water bonding with defect sites [52,53].

^1^H MAS NMR spectrum of the sample with ibuprofen prepared in a water-free environment with the host dehydrated at 500 °C is presented in Figure S6 (Supplementary Information). Narrow signals from mobile ibuprofen are still present, so this phenomenon is not connected directly with the presence of water in a zeolite sample.

**Figure 8:**
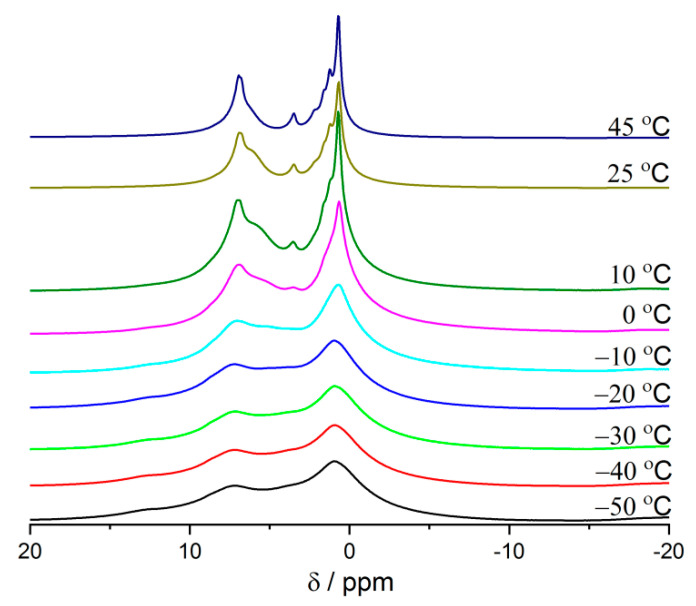
^1^H MAS NMR spectra of sample FAU31–24IBU recorded at different temperatures.

The second hypothesis was connected to a long time that ibuprofen needs to cool down in the sample and to equilibrate in the complicated porous architecture of a mesoporous zeolite. NMR experiments performed at different temperatures from −50 °C to 45 °C show that the lines in ^1^H MAS NMR spectra are getting narrower with an increase of the temperature for the sample FAU31-24IBU (Figure 8). This shows that ibuprofen is very mobile in this sample when it is heated to 45 °C.

## 4. Discussion

High mobility in a dehydrated state was observed in the samples treated in an oven for an overnight treatment (Figure 5A(c),B(c),C(c)) and in the samples prepared in a dehydrated state (Figure 6 and Figure 7A(a),B(a),C(a)). Special care was taken that the rotors were not transported to NMR spectrometer right after thermal treatments so that a sample had the time to cool down and equilibrate in a water-free desiccator for at least 20 min (for an oven treatment) and 30 min in a glove box (for the water-free synthesis). Despite that, increased mobility was observed even after 3 h after thermal treatments. What is more, this phenomenon is probably not connected with molecules located in mesopores. On the contrary, when the ibuprofen content was up to 24%, for the samples without water, this phenomenon was not observed (Figure 5A(a,b) and Figure 6a–c). Therefore, this feature is ascribed to ibuprofen molecules located in intercrystalline voids. That is why in a 4 mm rotor these liquid-like signals from ibuprofen were much more intense (Figure 7) than in a smaller rotor that contained a smaller amount of sample (Figure 6). Probably, ibuprofen in a closed rotor needs much more time to cool down and crystallize. For at least few hours after the heating treatment, ibuprofen stays in a liquid phase in between the voids between crystals. Only after a prolonged time, the signals characteristic for ibuprofen with low mobility located outside mesopores is present (carbon 3 in Figure 7B(b),C(b)). It is also probable that a high rotation speed (10 kHz) and high power decoupling used in ^13^C CP MAS NMR experiments may influence the temperature of the sample and, in effect, elongate the effect of liquification of ibuprofen in the samples.

## 5. Conclusions

Ibuprofen introduced into mesopores of dealuminated zeolite Y exists in a similar state as in other mesoporous materials. It does not form crystals but exists in a mobile phase with considerable shifts of the melting and evaporation temperatures. Solid-state NMR characterization of the zeolite samples with ibuprofen in ambient conditions, after hydration and dehydration proved that water increases the resolution of ^1^H MAS NMR spectra, and thus the mobility of the introduced phase. Surprisingly, the increased mobility of ibuprofen was also observed in a dehydrated state. This was ascribed to organic species located outside mesopores that were still very mobile after the thermal treatment. It took more than 3 h for ibuprofen to equilibrate in water-free conditions in a closed rotor. These observations are important for the preparation of guest–host systems using the melting method and the use of zeolites in drug delivery. Possibly, the impact of water on the mobility of the pharmaceuticals in drug delivery systems could lead to the modification of the delivery methods by the regulation of the humidity or temperature of the systems.

## Figures and Tables

**Table 1:** Mass % of ibuprofen, water, specific surface areas (S_BET_), pore diameters, and pore volumes of the samples under study.

Sample	Ibuprofen [%] ^1^	Water [%] ^2^	S_BET_ [m^2^/g]	Pore Diameter [nm] ^3^	Pore Volume [cm^3^/g]
FAU31	-	14.3	716	3.8	0.53
FAU31-12IBU	12.5	5.4	489	3.8	0.40
FAU31-24IBU	23.9	4.5	34	3.8	0.1
FAU31-38IBU	38.0	1.6	33	-	0.01

^1^ TG: 120–1000 °C, ^2^ TG: 30–120 °C; ^3^ from BJH desorption branch.

## Data Availability

Not applicable.

## Supplementary Materials

The following are available online at https://www.mdpi.com/article/10.3390/ma14247823/s1, Figure S1. Pore size distributions from BJH desorption branches recorded for Nitrogen Adsorption for the samples under study., Figure S2. TG curves for the samples under study. There are two regions of interest: from 30 to 120 °C (I) that is used for quantification of the water content, and from 121 to 1000 °C (II) that is used for the calculation of the ibuprofen content in the samples., Figure S3: Molecular structure of ibuprofen with labelled positions of atoms. The convention of numbering for protons and carbons is the same. There is no proton in positions 4, 9, and 1. The signals in ^1^H MAS NMR spectra labelled 1 comes from OH from the carboxyl group., Figure S4: ^1^H MAS NMR spectra of the sample prepared in a water-free environment. Colors denote the time that passed after the heating treatment in a closed rotor. The signals from ibuprofen molecules are very narrow and are getting even narrower with the time. The inset in a chosen region of chemical shifts between 5.5 and 8 ppm show clearly this tendency., Figure S5: ^1^H MAS NMR spectra of the parent zeolite FAU31 degassed in vacuum in 300 °C and in 500 °C. Arrows indicate signals from bonded water, at ca. 2.5 ppm and 6.2 ppm., Figure S6: ^1^H MAS NMR spectrum of the sample FAU31 degassed in 500^o^C with introduced ibuprofen molecules in a water-free environment.
