# Supplementary material for: The Impact of Hydration and Dehydration on the Mobility and Location of Ibuprofen Molecules in the Voids of Ultra-Stable Zeolite Y
Source: Materials (Basel). 2021 Dec 17;14(24):7823. doi: 10.3390/ma14247823 (PMC8709391; doi:10.3390/ma14247823)
Supplement: Supplementary file 1 [file materials-14-07823-s001.zip › materials-1489457-supplementary.pdf]

Supplementary Information

The impact of hydration and dehydration on the mobility and location of ibuprofen molecules in the voids of ultra-stable zeolite Y

Mariusz Gackowski [1]* and Mateusz Paczwa [2]

[1] Jerzy Haber Institute of Catalysis and Surface Chemistry Polish Academy of Sciences; ncgackow@cyf-kr.edu.pl
[2] Institute of Physics, University of Szczecin, mateusz.paczwa@usz.edu.pl
* Correspondence: ncgackow@cyf-kr.edu.pl

Figure S1……………………………………………………………………... S2
Figure S2……………………………………………………………………… S2
Figure S3……………………………………………………………………… S3
Figure S4……………………………………………………………………… S3
Figure S5……………………………………………………………………... S4
Figure S6……………………………………………………………………… S5

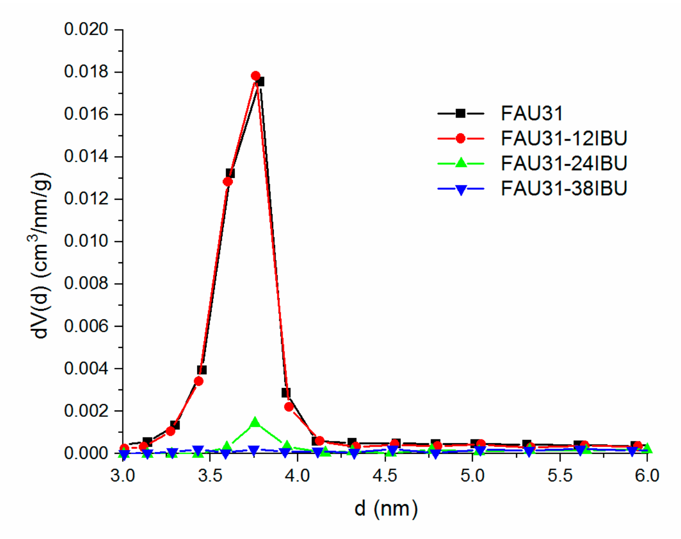

Figure S1. Pore size distributions from BJH desorption branches recorded for Nitrogen Adsorption for the samples under study.

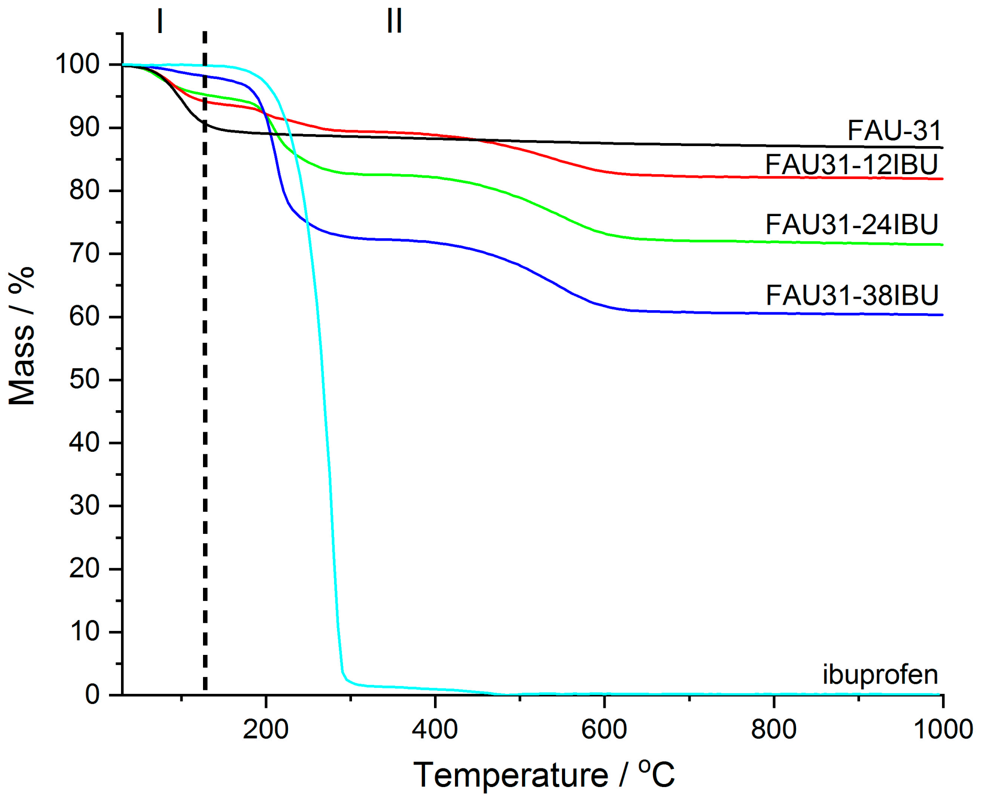

Figure S2. TG curves for the samples under study. There are two regions of interest: from 30 to 120 °C (I) that is used for quantification of the water content, and from 121 to 1000 °C (II) that is used for the calculation of the ibuprofen content in the samples.

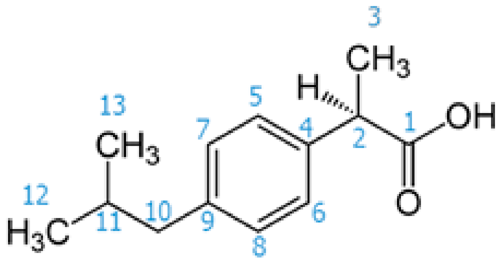

Figure S3. Molecular structure of ibuprofen with labelled positions of atoms. The convention of numbering for protons and carbons is the same. There is no proton in positions 4, 9, and 1. The signals in ^{1}H MAS NMR spectra labelled 1 comes from OH from the carboxyl group.

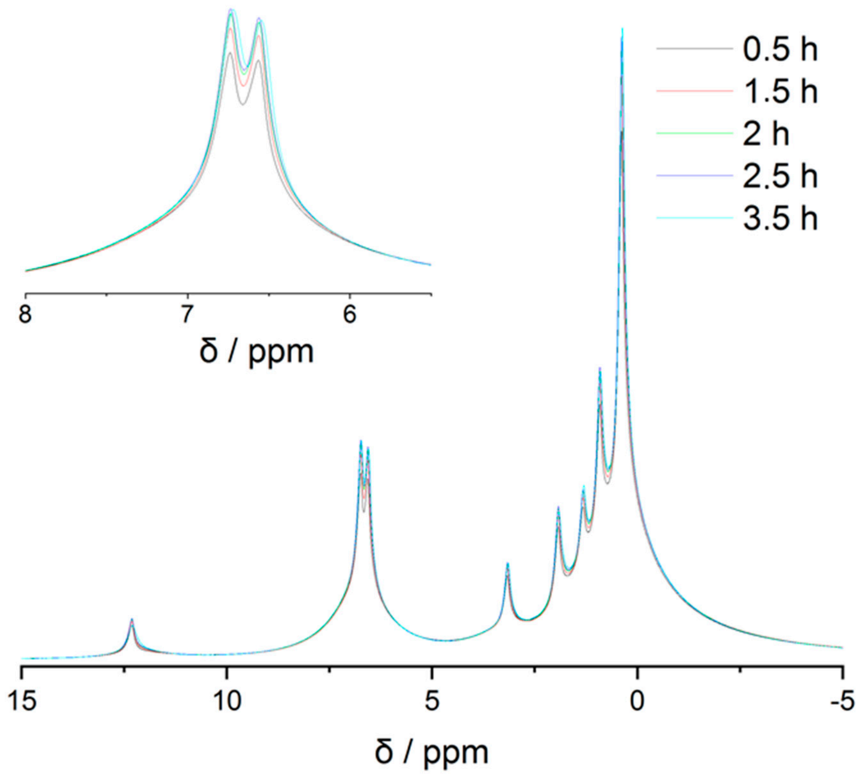

Figure S4. ^{1}H MAS NMR spectra of the sample prepared in a water-free environment. Colors denotes the time that passed after the heating treatment in a closed rotor. The signals from ibuprofen molecules are very narrow and are getting even narrower with the time. The inset in a chosen region of chemical shifts between 5.5 and 8 ppm show clearly this tendency.

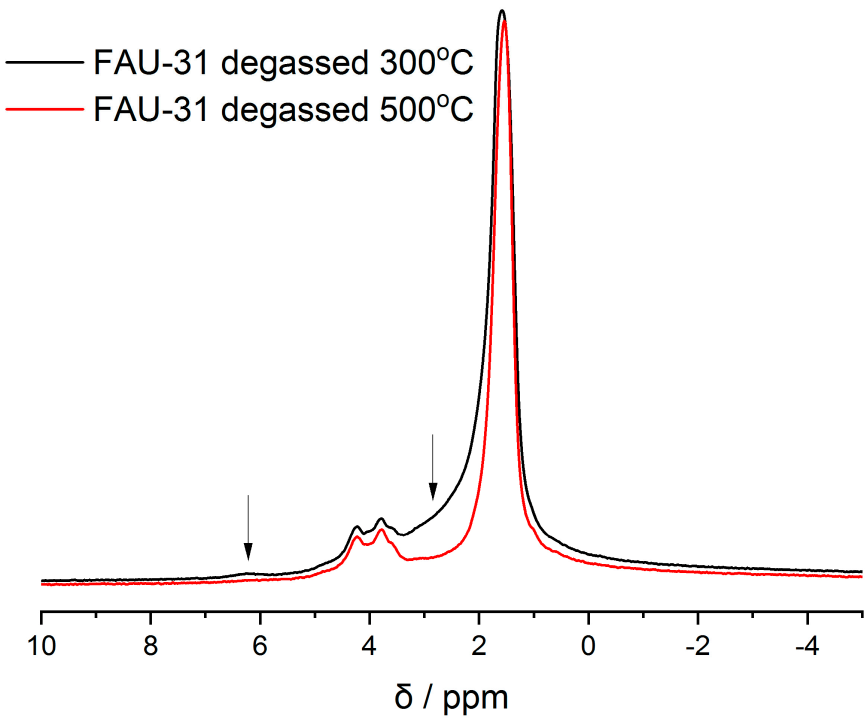

Figure S5. ^{1}H MAS NMR spectra of the parent zeolite FAU31 degassed in vacuum in 300 °C and in 500 °C. Arrows indicate signals from bonded water, at ca. 2.5 ppm and 6.2 ppm.

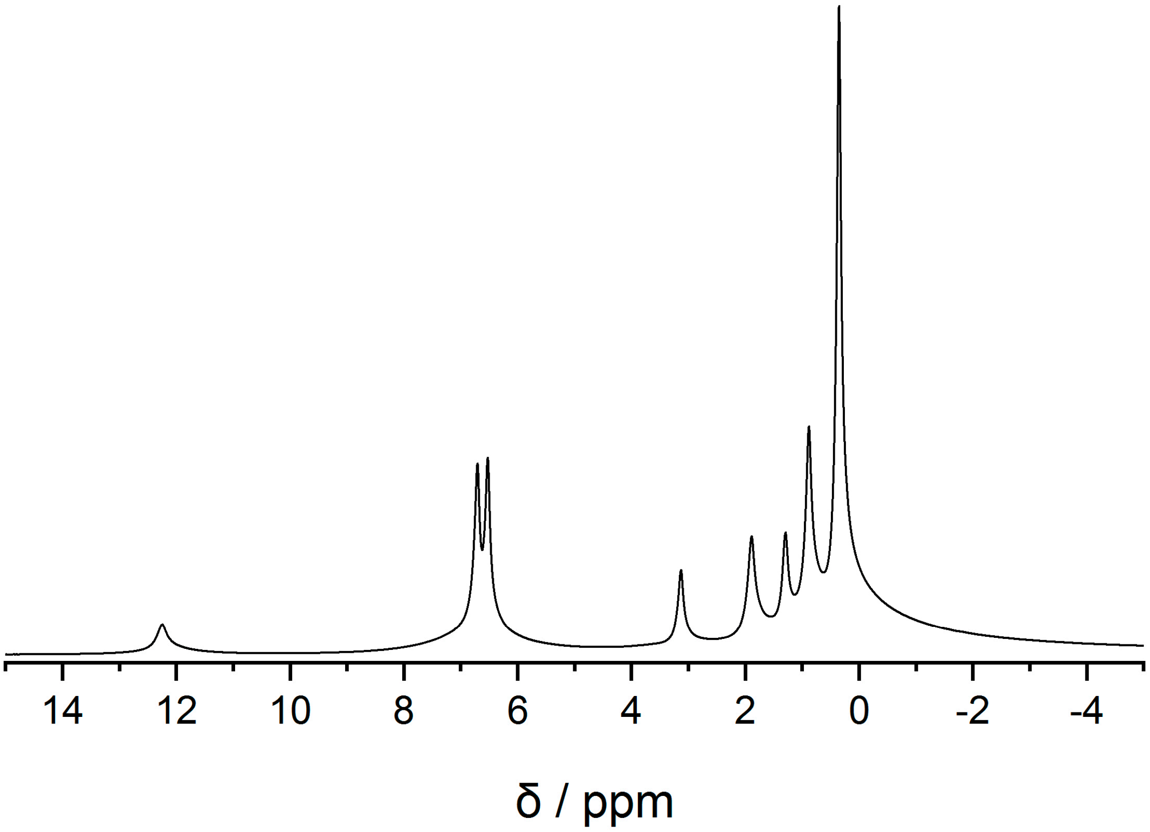

Figure S6. ^1H MAS NMR spectrum of the sample FAU31 degassed in 500°C with introduced ibuprofen molecules in a water-free environment.
